# Study of Amiloride Binding to Human Serum Albumin: Insights from Thermodynamic, Spectroscopic, and Molecular Docking Investigations

**DOI:** 10.3390/molecules28237688

**Published:** 2023-11-21

**Authors:** Safikur Rahman, Sana Iram, Md Tabish Rehman, Afzal Hussain, Arif Tasleem Jan, Jihoe Kim

**Affiliations:** 1 Munshi Singh College, BR Ambedkar Bihar University, Muzaffarpur 845401, India; shafique2@gmail.com; 2 Department of Medical Biotechnology, Yeungnam University, Gyeongsan 712-749, Republic of Korea; sanairam157@yu.ac.kr; 3 Department of Pharmacognosy, College of Pharmacy, King Saud University, Riyadh 11451, Saudi Arabia; mrehman@ksu.edu.sa (M.T.R.); afihussain@ksu.edu.sa (A.H.); 4 School of Biosciences and Biotechnology, Baba Ghulam Shah Badshah University, Rajouri 185234, India; atasleem@bgsbu.ac.in

**Keywords:** amiloride, FRET, protein–ligand interaction, human serum albumin

## Abstract

This study was undertaken to investigate the interaction between the sodium channel blocker amiloride (AML) and human serum albumin (HSA). A combination of multi-spectroscopic techniques and computational methods were employed to identify the AML binding site on HSA and the forces responsible for the formation of the HSA–AML complex. Our findings revealed that AML specifically binds to Sudlow’s site II, located in subdomain IIIA of HSA, and that the complex formed is stabilized using van der Waals hydrogen-bonding and hydrophobic interactions. FRET analysis showed that the distance between AML and Trp214 was optimal for efficient quenching. UV-Vis spectroscopy and circular dichroism indicated minor changes in the structure of HSA after AML binding, and molecular dynamics simulations (MDS) conducted over 100 ns provided additional evidence of stable HSA–AML-complex formation. This study enhances understanding of the interaction between AML and HSA and the mechanism responsible.

## 1. Introduction

Human serum albumin (HSA) (molecular weight-66 kDa) is the most abundant protein in human plasma. This heart-shaped transporter plasma protein is produced in the liver, is soluble in water, and consists of 585 amino acids arranged in a single polypeptide chain folded into three structurally homologous domains (domains I, II, and III), each composed of two subdomains (A and B) [1]. The physiochemical and structural properties of HSA are well established [2,3]. HSA is rich in α-helices, is stabilized with 17 disulfide bonds, and a single tryptophan (Trp214) residue located in a hydrophobic cavity in subdomain IIA that is used as a probe in spectroscopic studies. The binding of HSA with different ligands is a topic of considerable interest in the biochemistry and pharmacology fields [4,5]. HSA extends the shelf life of drugs by protecting them while they undergo metabolism and elimination in vivo, thereby contributing to their pharmacokinetic properties. Being biocompatible and non-immunogenic, it exhibits the property of being exploited as an appealing carrier in drug delivery. Therefore, HSA is used to transport a wide range of endogenous and exogenous molecules, including xenobiotics, drugs, and hormones, reduce drug toxicity, increase drug solubility in plasma, and protect drugs from oxidation [6]. The interactions between drugs and HSA critically influence ADME properties (absorption, distribution, metabolism, and excretion) and prevent undesirable drug–drug interactions [7,8]. Thus, many studies have been conducted to determine the nature of interactions and binding sites of various HSA ligands, such as ibuprofen (anti-inflammatory drug), and erucic acid (fatty acid) binds to Sudlow’s site II of HSA [9,10], and cyclobenzaprine hydrochloride (muscle relaxant) and warfarin (anticoagulant) bind to Sudlow’s site I [11,12].

Amiloride hydrochloride (AML) is a substituted pyrazine-carbonyl-guanidine administered orally with other diuretics (thiazide, frusemide, etc.) to control potassium levels in patients with hepatic cirrhosis, hypertension, or who are at risk of hypokalemia [13]. AML is an acid-sensing, ion channel inhibitor [14] that acts on distal tubules of nephrons to inhibit sodium–potassium exchange. In addition, AML reportedly blocks Na^+^/H^+^ antiporter and Na^+^/Ca^+^ exchanger in cardiac myocytes and other epithelial cells [15,16,17], and biochemical, cellular, and in vivo studies suggest AML has antitumor and antimetastatic effects [18,19]. Moreover, by competitively inhibiting uPA (urokinase-type plasminogen activator), AML suppresses the invasion and metastasis of cancer cells [20,21]. Interestingly, a synergistic effect was observed on breast cancer cell apoptosis when AML analogue 5-N, N-dimethylamiloride was co-administered with a combination of paclitaxel [22]. A recent study showed that long-term, low-dose AML effectively maintained micro- and macro-circulatory functions and controlled blood pressure in patients suffering from hyperaldosteronism [23]. However, despite many studies on the pharmacological effects of AML, its binding to HSA has not been explored.

In the present study, the interaction between HSA and AML was comprehensively studied using various spectroscopic and in silico methods. Various thermodynamic parameters and fluorescence-quenching experiments were used to investigate the nature of HSA to AML binding. AML-induced changes in the secondary and tertiary structures of HSA were monitored using circular dichroism and UV-Vis spectroscopic techniques, respectively. Changes in the amino acid microenvironment of HSA caused by drug binding were also investigated using synchronous and 3D fluorescence techniques. In addition, binding mechanisms and residue-interaction patterns were investigated using molecular docking (MD) and molecular dynamics simulation (MDS) studies.

## 2. Results and Discussion

### 2.1. Investigating the Interaction between HSA and AML

#### 2.1.1. Fluorescence-Quenching Analysis

**Figure 1:**
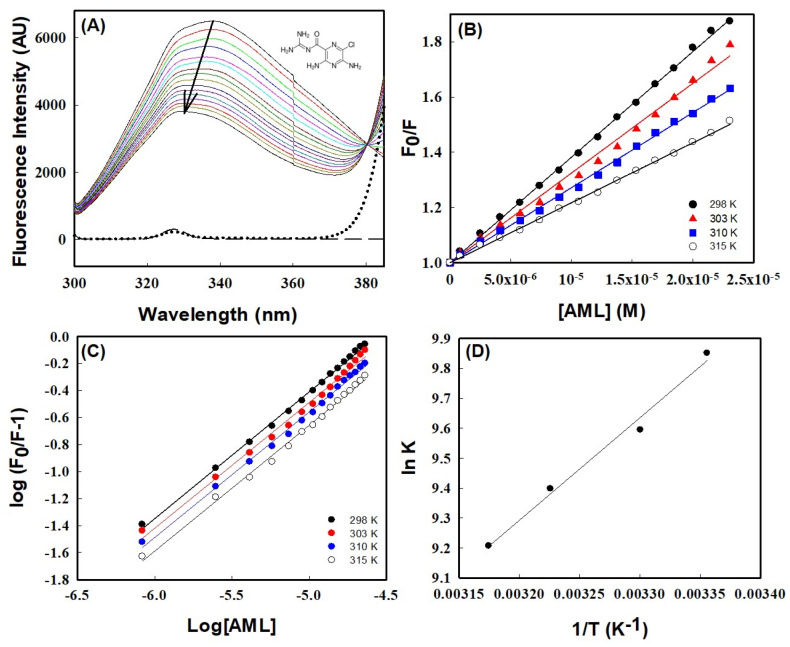
Fluorescence quenching with AML binding to HSA. (**A**) Fluorescence emission spectra of HSA (4 μM) and at 298 K in the absence and the presence of increasing concentrations of AML (colored solid lines), buffer (long dashed line), and AML alone (dotted line). The structure of AML is shown in the inset. The arrow indicates fluorescence quenching by increasing AML concentrations; (**B**) Stern–Volmer plots of HSA quenching with AML at different temperatures; (**C**) plot of log (*F_o_/F*)/*F* vs. log [*Q*] (the modified Stern–Volmer plot); and (**D**) Van ’t Hoff plot of HSA and AML interactions, which was used to calculate thermodynamic parameters.

The binding of AML to HSA was investigated by measuring changes in HSA intrinsic fluorescence in the presence or absence of AML at various temperatures (298, 303, 310, and 315 K). The intrinsic fluorescence-emission spectrum of HSA when excited at 295 nm is primarily due to Trp214 residues [24]. In the absence of AML, HSA showed strong emission peaks at 340 nm and 298 K, whereas in the presence of HSA gradual quenching was observed as AML concentrations increased in the range 0–25 µM (Figure 1A). Notably, AML was non-fluorescent in the range where Trp emission was observed (Figure 1A, dotted line); therefore, the decrease in fluorescence intensity can be ascribed to the quenching of HSA fluorescence by AML. The quenching of Trp emission may have occurred because of the proximity of the quencher to the fluorophore. The fluorescence quenching of HSA by AML was compared with the quenching by the already-reported drug CBH to ensure whether the fluorescence intensity decreased because of the quenching of Trp fluorescence [25]. From Figure 1, it was clear that the quenching of HSA fluorescence was because of binding of AML to HSA. A significant blue shift (10 nm—from 339 nm to 329 nm) in fluorescence intensity was also observed when increasing the concentration of AML to 25 µM. The shift in emission maximum may have resulted from a modification in the polarity of Trp residues (toward a more hydrophobic environment). The observed quenching and blue shift confirmed the association between AML and HSA. Furthermore, fluorescence experiments at temperatures other than 298 K showed similar patterns, i.e., a progressive decrease in emission and blue shifting with increasing AML concentration. The observed quenching and blue shift confirmed the association between AML and HSA. Furthermore, fluorescence experiments at temperatures other than 298 K showed similar patterns, i.e., a progressive decrease in emission and blue shift with increasing AML concentration.

There are two mechanisms involved in ligand-driven fluorescence quenching, usually classified as static and dynamic, which can be differentiated using their temperature-dependent behaviors [26]. To elucidate the binding mechanism, fluorescence titration data were investigated using the Stern–Volmer equation.

(1) $$\frac{F_{o}}{F}=1+K_{SV}\left[Q\right]=1+k_{q}\tau_{o}[Q]$$

In this equation, *F_o_* and *F* denote the intensity of HSA fluorescence emission with or without AML, and [Q] represents the quencher concentration (i.e., AML concentration). *K_SV_* is the Stern–Volmer quenching constant; *τ_o_* is the fluorescence lifetime of HSA in the excited state in the absence of a quencher (its value is ~10^−8^ s); and *k_q_* is the protein bimolecular-quenching-rate constant. Figure 1B shows Stern–Volmer plots (*F_o_/F* versus [*Q*]) at different temperatures, which were used to obtain *K_SV_* values. Table 1 shows the values of *K_SV_* and other parameters. The magnitude of *K_SV_* obtained at 298 K was 3.82 × 10^4^ M^−1^, which is in excellent agreement with earlier reports for HSA drug interactions for many drugs (values range from 10^4^ to 10^6^ M^−1^ [10,27,28]. Furthermore, a linear Stern–Volmer plot within the investigated concentrations range (0–25 µM) indicated a single quenching mechanism (Figure 1B). Table 1 confirmed that the quenching mechanism involved was static, as the value of *K_SV_* decreased with increasing temperature, indicating dissociation of HSA–AML complex at higher temperatures due to a large diffusional coefficient and a reduction in complex stability. These observations were further supported by the bimolecular quenching rate constant (*k_q_*) calculated from *K_SV_*, i.e., *k_q_* = *K_SV_*/*τ_o_*. The obtained *k_q_* values fell in the range of 6.6–3.75 × 10^−12^ which were much higher than the diffusion constant (*k_q_* values ~10^10^ M^−1^ s^−1^), further confirming that the quenching mechanism is static during complex formation. The *k_q_* and *K_SV_* values suggest that the quenching mechanism was static, involving ground-state complex formation between HSA and AML at equilibrium.

#### 2.1.2. Evaluations of Thermodynamic and Binding Parameters

The binding between HSA and ligands is stabilized through diverse non-covalent interactions, such as hydrophobic forces, hydrogen bonds, electrostatic interaction, and van der Waal interactions. The nature of forces associated with HSA–AML complex formation can be predicted from the thermodynamic parameters of the binding process. So, it is essential to understand the thermodynamic parameters of binding between HSA and AML. In particular, the binding constant, which represents equilibrium between free and bound molecules, is essential for calculating thermodynamic parameters. The modified Stern–Volmer equation was used to determine two parameters of AML to HSA binding, namely, (i) the number of binding sites (*n*) and (ii) the binding constant for static quenching (*K_b_*).

(2) $$log\left(\frac{F_{o}-F}{F}\right)=logK_{b}+nlog[Q]$$

The plot of double logarithms, i.e., log [(*F_o_* − *F*)/*F*] vs. log [*Q*] in Figure 1C provided the value of binding stoichiometry, which was in the range 0.92–0.93 for all temperatures studied (Table 1), suggesting a single binding-site interaction between HSA and AML. However, binding constant values were varied with temperature, i.e., 1.89× 10^4^ M^−1^ at 298 K to 0.99 × 10^4^ M^−1^ at 315 K. From Table 1, it is clear that temperature has a similar effect on the binding constant and Stern–Volmer’s constant, viz. Both decreased when the temperature increased. Earlier reports suggested the binding constants of several HSA ligands vary between 10^2^ and 10^6^ M^−1^, indicating weak to strong binding [29,30,31,32]. However, we found the binding constant for the AML to HSA interaction was 1.89 × 10^4^ at 298 K, revealing a significant interaction between AML and HSA. The type of interaction between HSA and AML can be determined by analyzing thermodynamic parameters of binding, such as entropy (∆*S*°), enthalpy change (∆*H*°), and Gibb’s free-energy change (∆*G*°). To obtain the above thermodynamic parameters (∆*H*° and ∆*S*°) for the interaction between HSA and AML, we used the Van ’t Hoff equation (Equation (6)) and assumed ∆*H*° was unperturbed in the studied temperature range.

(3) $$InK_{b}=\left(\frac{-∆H^{{}^{\circ}}}{R}\right)\frac{1}{T}+\frac{∆S^{{}^{\circ}}}{R}$$

where *K_b_* is the temperature-dependent binding constant (in Kelvin), and *R* is the universal gas constant. The slope of the linear Van ’t Hoff plot and intercept (ln*K_b_* vs. 1/*T*) gave values for ∆*S*°/*R* and −∆*H*°/*R*, respectively (Figure 1D). The values ∆*H*° and *T*∆*S*°, thus obtained, are listed in Table 1. According to the theory of Ross and Subramanian, negative signs of ∆*H*° (−6.80 kcal mol^−1^) and *T*∆*S*° (−0.98 kcal mol^−1^) at 298 K indicate that the HSA to AML interaction was due to van der Waals forces and hydrogen bonds [33]. In addition, ∆*G*° was calculated using ∆*H*° and *T*∆*S*° values using Equation (4),

(4) $${\;∆G}^{{}^{\circ}}=∆H^{{}^{\circ}}-T∆S^{{}^{\circ}}$$

The calculated value of ∆*G*° was −5.81 kcal mol^−1^ at 298 K (Table 1). ∆*G*° values at other temperatures are listed in Table 1. The negative sign of ∆*G*° indicated that complex formation between AML and HSA is a favorable process.

#### 2.1.3. Förster Resonance Energy Transfer (FRET) between HSA and AML

**Figure 2:**
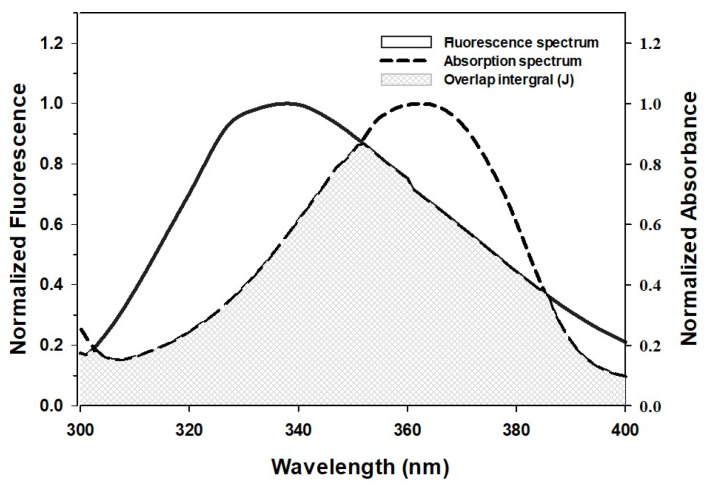
Spectral overlap of the fluorescence-emission spectrum of HSA and the absorption spectrum of AML.

FRET is a nondestructive spectroscopic technique used to assess the proximity of donor and acceptor molecules, and the level of overlap between the emission and absorption spectra of the donor and acceptor, respectively, determines FRET efficiency. In addition, donor quantum yield, distance, and transition dipole orientation of donor and acceptor also affect FRET [34]. For efficient FRET, the donor/acceptor distance should be no more than 7 nm. Figure 2 shows that the emission spectrum of HSA, and the absorption spectrum of AML overlap, which indicates the possibility of FRET.

During FRET, energy from the excited-state donor fluorophore (Trp214 in the case of HSA) is transferred to the acceptor molecule. This energy-transfer efficiency can be used to determine the distance (*r*) between the AML and Trp214 of HSA. Equation (5) can be used to calculate FRET efficiency, which is inversely proportional to the sixth power of the distance between the HSA donor fluorophore and AML.

(5) $$E=\frac{R_{o}^{6}}{R_{o}^{6}+r_{o}^{6}}=1-\frac{F}{F_{o}}$$

where *F_o_* and *F* are the intensities of the fluorescence of the donor molecule (HSA) in the absence and presence of the acceptor (AML), the distance between acceptor and donor molecules is denoted by *r*. Furthermore, *R_o_* is the critical distance between acceptor and donor molecules at which energy-transmission efficiency becomes 50%. The following equation is used to obtain values of *R_o_.*

(6) $$R_{o}^{6}=8.79\times 10^{-25}K^{2}n^{-4}\Phi J$$

where *K*^2^ specifies the orientation factor of acceptor and donor dipoles, *n* is the average refractive index of medium, Φ is the quantum yield of donor fluorescence, and *J* is the overlap area between the fluorescence emission spectrum of the donor and the absorption spectrum of the acceptor.

*J* is defined as

(7) $$J=\frac{\int_{0}^{\infty }F_{\lambda }\varepsilon_{\lambda }\cdot d\lambda }{\int_{0}^{\infty }F_{\lambda }\cdot d\lambda }$$

where the fluorescence intensity of the donor at wavelength *λ* is *F_λ_*, and the molar extinction coefficient of the acceptor at *λ* is *ε_λ_*.

The values of Φ, *K*^2^, and *n* (0.118, 2/3, and 1.33, respectively) were adopted from a previous study [33]. *R_o_*, *J*, and *r* were estimated to be 2.26 nm, 5.99 × 10^−15^ M^−1^ cm^3^, and 2.33 nm, respectively (Table 2). Notably, *R_o_* and *r* values were in the 2–8 nm range, thus confirming the requirement for FRET. Further, the *r* value was within the 0.5 *R_o_* < *r* < 1.5 *R_o_* window, which is in accordance with Förster’s theory and also indicates a static quenching mechanism observed for HSA and AML interaction [34].

#### 2.1.4. Location of the AML Binding Site in HSA

**Figure 3:**
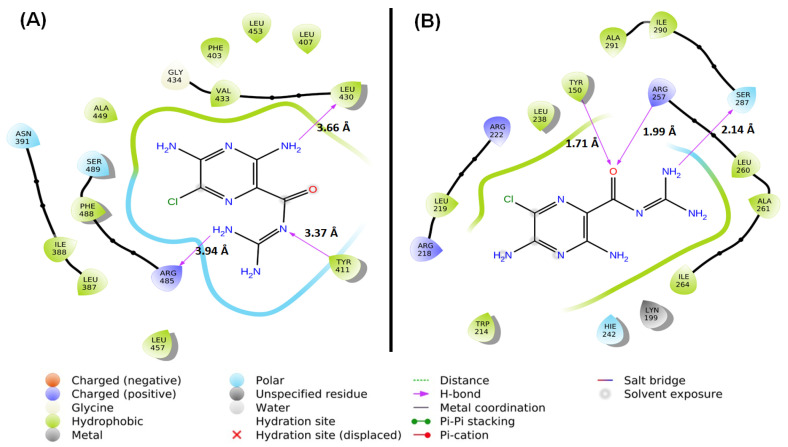
MD between HSA and AML at different binding sites. (**A**) Sudlow’s site I (subdomain IIA) and (**B**) Sudlow’s site II (subdomain IIIA).

MD studies were carried out to determine the location of AML binding and to identify the residues participating in the interaction between AML and HSA. Docking analysis revealed that AML primarily binds to subdomain IIIA (known as Sudlow’s site II) of HSA and exhibits lesser affinity for subdomain IIA (Sudlow’s site I) (Figure 3).

Within subdomain IIIA, AML exhibited specific interactions (hydrophobic, polar, etc.) with several residues, namely Ser489, Phe488, Arg485, Leu457, Leu453, Ala449, Gly434, Val433, Leu430, Tyr411, Leu407, Phe403, Asn391, Ile388, and Leu387 (Figure 3A). Notably, AML formed an H-bond with each of the three residues, Tyr411 (3.37 Å), Leu430 (3.66 Å), and Arg485 (3.94 Å). At subdomain IIIA, AML formed hydrophobic interactions with Leu387 (4.55 Å), Ile388 (4.14 Å), Val433 (4.28 Å), and Leu453 (5.38 Å). Hence, hydrogen bonding and hydrophobic interactions stabilized HSA–AML at Sudlow’s site II in subdomain IIIA. In contrast, at Sudlow’s site I (subdomain IIA), the interaction between HSA and AML involved Leu238, Arg222, Leu219, Arg218, Trp214, Lys199, Tyr150, Hie242, Ala291, Ile290, Ser287, Ile264, Ala261, Leu260, and Arg257 (Figure 3B), and AML interacted with HSA by hydrogen bonding with Tyr150 (1.71 Å), Arg257 (1.99 Å), and Ser287 (2.14 Å). From an earlier report, it was suggested that Trp214 contributes to drug stabilization in Sudlow’s site I [35]. In addition, hydrophobic interactions between HSA and AML at subdomain IIA were formed by Phe211 (5.84 Å), Leu238 (4.34 Å), and Hie242 (5.43 Å). Hydrogen bonds and hydrophobic interactions play important roles in the stabilization of HSA–AML complex in HSA subdomain IIA. The docking energy (XP Glide score) of the AML/HSA interaction at Sudlow’s site I (subdomain IIA) and Sudlow’s site II (subdomain IIIA) were estimated to be −6.15 and −9.75 kcal/mol, respectively. It is important to mention here that most of the ligands bind primarily with Sudlow’s site I and II binding site, in which Sudlow’s site I has a large binding pocket as compared to Sudlow’s site II [8,36]. In addition, warfarin (anticoagulant), Galantamine (AChE inhibitor), Bulky heterocyclic anions, etc., binds to site I, and ibuprofen (non-steroidal anti-inflammatory drug), propofol (general anesthetic), and aromatic carboxylates with an extended conformation prefer Sudlow’s site II [37].

### 2.2. Effect of AML on the Conformation of HSA

#### 2.2.1. UV-Vis Spectroscopy

**Figure 4:**
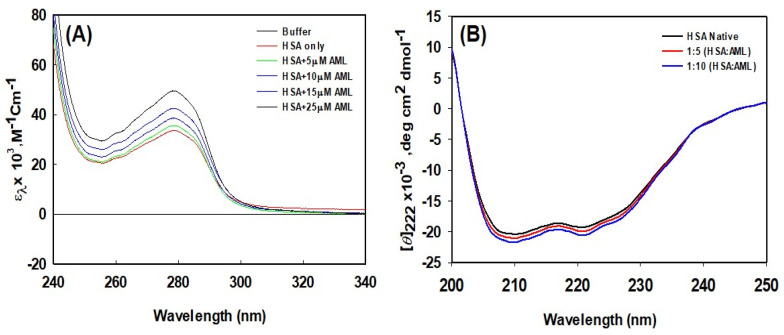
HSA conformational alterations caused by AML binding. (**A**) UV-Vis absorption spectra of HSA in the absence or presence of different concentrations of AML, and (**B**) far-UV CD spectra of HSA in the absence or presence of AML (values shown are molar ratios).

UV-Vis spectroscopy is commonly used to observe changes in protein conformation caused by ligand interactions. The UV-Vis absorption spectra of HSA in the presence and absence of different concentrations of AML are shown in Figure 4A, which shows plots of molar absorptivity versus wavelength. In the absence of AML, the UV-Vis absorption spectra of HSA exhibited a significant peak at 278 nm, which is a characteristic feature of a properly folded globular protein. Upon increasing the concentration of AML, the wavelength at maximum absorption did not alter significantly. On the other hand, the strength of the signal at 278 nm increased with increasing AML concentration, and at an AML concentration of 25 µM, the peak intensity at 278 nm increased by ~35% versus HSA absorbance without AML. Thus, AML binding increased the molar absorptivity of HSA changes, indicating that AML altered the environment around aromatic amino acids.

#### 2.2.2. Analysis of Secondary Structural Changes Using Circular Dichroism (CD) in the Far-UV

CD spectroscopy is a technique used to explore the tertiary and secondary structures of proteins. Figure 4B shows the far-UV CD spectra of HSA and HSA–AML complexes. Two negative peaks were observed at 222 and 209 nm, which are characteristic of the α-helical nature of HSA. Molar residue ellipticity (MRE) values in the absence of AML concur with previous reports [38]. The change in the far-UV CD spectrum of HSA after the addition of AML indicates a change in the secondary structure of HSA due to AML binding. Changes in the secondary-structure contents (i.e., changes alpha and beta compositions) produce structural changes in proteins, depriving them of biological activity. Several previous investigations have also found that structural changes in HSA occur as a result of drug or ligand interactions, for example, the binding of the 2,4-thiazolidinedione, cyclobenzaprine hydrochloride, eperisone hydrochloride, erucic acid, and imipenem [10,11,33,38,39]. These interactions cause significant secondary structural alterations in HSA.

#### 2.2.3. Analysis of Tertiary Structure Changes Using Synchronous Fluorescence

**Figure 5:**
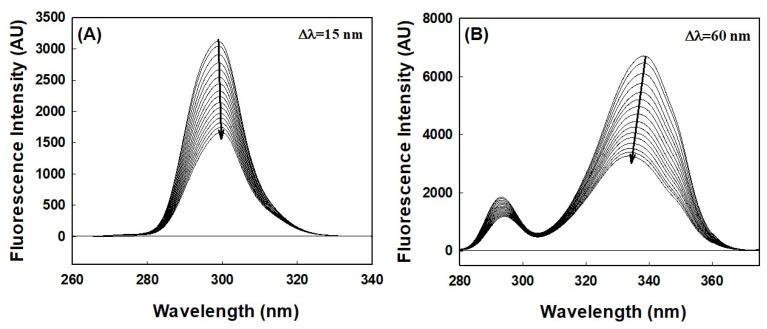
HSA–synchronous fluorescence spectra in the absence or presence of different concentrations of AML; (**A**) Tyr (∆λ = 15 nm), and (**B**) Trp (∆λ = 60 nm) residues.

Synchronous fluorescence spectroscopy provides an excellent means of investigating aromatic amino acid microenvironments. Characteristic information about Tyr and Trp residue microenvironments can be retrieved by setting the scanning interval (Δλ) to 15 and 60 nm, respectively. Synchronous spectra of HSA in the absence and presence of AML are shown in Figure 4. When the wavelength interval was set at 15 nm, no blue or red shift in maximum HSA emission was observed with increasing AML concentration, indicating that the microenvironment around Tyr had not changed (Figure 5A). However, when the wavelength interval was maintained at 60 nm, blue shifts (from 338 to 334 nm) were observed with increasing AML concentration, which showed that AML created a more hydrophobic environment around Trp214 and is less exposed to the solvent upon ligand attachment (Figure 5B).

#### 2.2.4. Three-Dimensional Fluorescence Analysis

**Figure 6:**
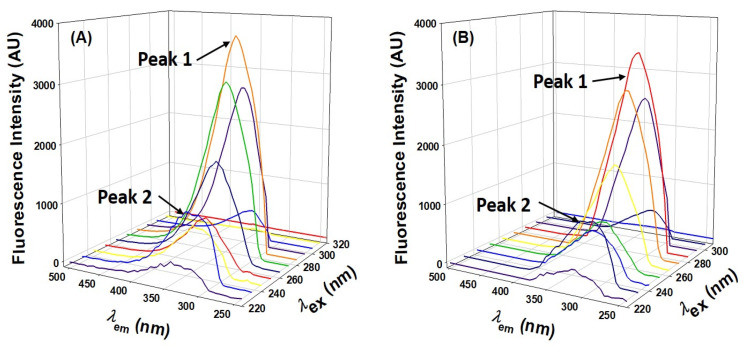
Three-dimensional HSA fluorescence spectra in the presence and absence of HSA. (**A**) HSA only; (**B**) HSA: AML at a molar ratio of 1:5.

Three-dimensional fluorescence spectroscopy is a powerful tool for characterizing protein conformational changes, and we utilized this technique to confirm AML-induced conformational changes. Three-dimensional fluorescence spectra of HSA and HSA–AML complexes had two distinct peaks (Figure 6). Peak I (ex = 280 nm) represented the structural characteristics of the backbone of the polypeptide caused by π→, π * transition of C=O and revealed conformational alterations. In contrast, peak II (λex = 225 nm) was associated with the spectral behavior of Tyr and Trp residues as a result of π → π * transition and reflects protein polarity in its microenvironment. Table 3 shows the spectral characteristics of HSA in the absence and presence of AML. The intensities of peaks 1 and 2 decreased upon adding AML, showing the perturbation in microenvironment around Tyr and Trp (Table 3 and Figure 6). Based on these findings, we concluded that, while the basic skeleton of HSA does not change considerably, when AML binding occurs, the polypeptide skeleton is disrupted, and the environment surrounding the amino acid residues of HSA is altered.

### 2.3. Molecular Dynamics Simulation

#### 2.3.1. Root-Mean-Square Deviation (RMSD)

**Figure 7:**
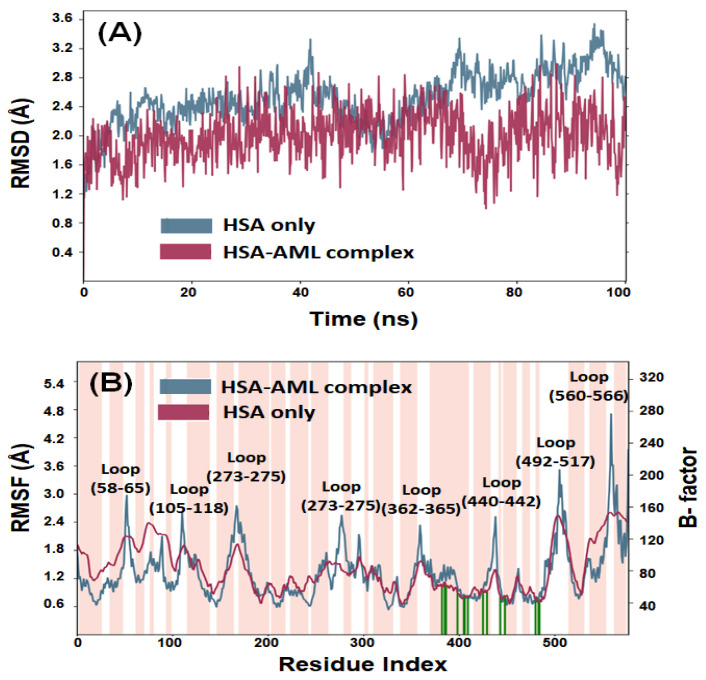
MDS of HSA–AML. (**A**) Root-mean-square deviations (RMSDs), and (**B**) root-mean-square fluctuations (RMSFs). The pink bar in panel B represents alpha-helical content of HSA, while white bars indicate the presence of loop. Different loop positions are also indicated. The green vertical lines on the *x*-axis represent the position of amino acid residues forming an interaction with the ligand.

An MDS study was conducted to assess the dynamic nature of the interaction between HSA and AML under physiological conditions and to confirm the stability of HSA–AML complex. The initial conformation of AML bound to HSA was used for a 100 ns MD simulation. Figure 7A illustrates the RMSDs of the Cα-atoms of HSA over the period of the MDS relative to the initial frame. A minor deviation in the RMSD of HSA alone was observed over the first 500 ps due to stabilization of the protein structure. However, after 500 ps, the system becomes stabilized and displays steady-state characteristics. Overall, the RMSD of HSA alone ranged from 1.6 Å to 3.4 Å, which was within the acceptable limit of 3.0 Å. Likewise, the RMSD of HSA–AML complex fluctuated insignificantly in the entire 0–100 ns MDS duration (Figure 7A). The RMSD value of the HSA–AML adduct ranged between 1.2 and 2.8 Å, suggesting the establishment of a stable complex. These findings imply that early changes in HSA and AML RMSDs were caused by the introduction of a substantial ligand into the hydrophobic cavity of HSA. However, when favorable contacts between HSA and AML had been established, a stable complex was produced during MDS.

#### 2.3.2. Root-Mean-Square Fluctuation (RMSF)

The root-mean-square fluctuations (RMSF) of all residues in HSA were determined by averaging the conformations obtained throughout the entire simulation. This analysis aimed to assess the dynamic behavior of the residues and provide insight into their structural flexibility and fluctuations. Generally, higher fluctuations indicate greater instability of the residues, while low RMSF values suggest stability. The RMSFs of HSA–AML complex (Figure 7B) were compared to the B-factor of HSA (determined during X-ray crystallography). The B-factor, also known as the temperature factor or Debye–Waller factor, is a measure of the thermal vibrations or disorder of atoms within a protein or a crystal structure. A high B-factor value indicates that the atoms in a particular region of the protein structure are highly disordered and have significant thermal motion. Conversely, a low B-factor value suggests that the atoms are relatively stable and have less thermal motion. Most of the residues in HSA–AML complex had RMSF values comparable with B-factor values of HSA alone, suggesting stability of the HSA–AML complex during MDS. However, some minor conformational changes were observed in the loop region and C-terminal residues, which tend to exhibit higher fluctuations. The slight variations in RMSF values may have been caused by the dynamic nature of ligands within the binding pocket.

#### 2.3.3. Radius of Gyration (rGyr) and Solvent-Accessible Surface Area (SASA)

**Figure 8:**
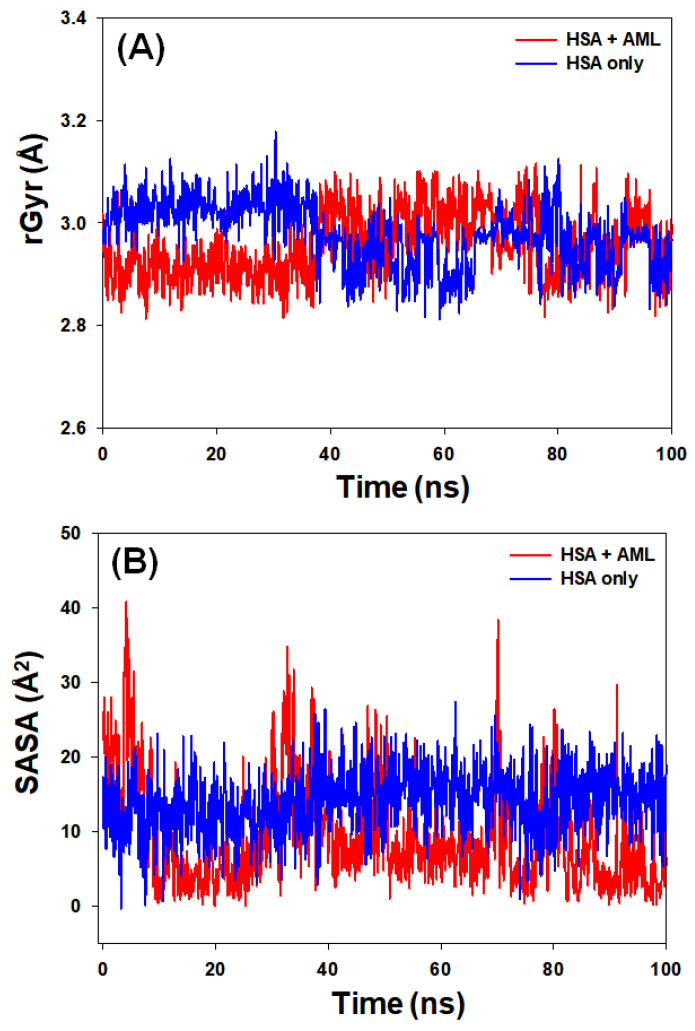
(**A**) Variations in the radius of gyration (rGyr) of HSA–AML complex and (**B**) solvent-accessible surface area (SASA) of HSA–AML complex as a function of simulation time.

The radius of gyration (rGyr) reflects the compactness of a protein–ligand complex, whereas solvent-accessible surface area (SASA) provides a measure of the exposure of a ligand–protein complex to the solvent molecules. These parameters offer insights into the stability of protein–ligand complexes during simulation. The analysis of rGyr revealed that there was a marginal decrease in the rGyr of HSA in the presence of AML. The values fluctuated in the range of 2.81–3.19 (mean value of 2.98 Å) for HSA alone and 2.81–3.08 Å (mean value of 2.93 Å) for HSA–AML complex (Figure 8A). Similarly, variations in the solvent-accessible surface area (SASA) of HSA alone and HSA–AML complex were examined to assess the exposure of amino acid residues to the solvent medium as a result of ligand binding (Figure 8B). The SASA of HSA alone and HSA–AML complex exhibited fluctuations within the ranges of 0–29 Å^2^ and 0–45 Å^2^ with mean values of 15.3 Å^2^ and 13.2 Å^2^, respectively. These results suggest that there was no significant change in the conformation of HSA due to the binding of AML, and the HSA–AML complex was stable in nature.

#### 2.3.4. Total Number of Contacts and Interacting Residues

**Figure 9:**
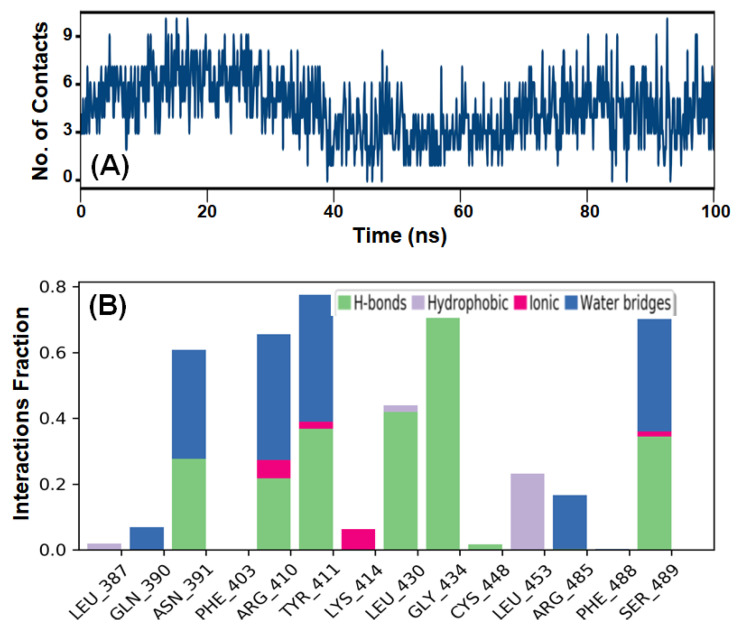
MDS results of the HSA-to-AML interaction. (**A**) The total number of contacts (hydrogen bonds, hydrophobic, ionic, and water bridges) formed between HSA and AML, and (**B**) intermolecular interactions between HSA and AML.

HSA–AML stability was investigated by evaluating the number of contacts established between HSA and AML during MDS. The total number of contacts formed was between zero and nine, and the average number of contacts between HSA and AML was four (Figure 9A). This observation complements the MD results that HSA-to-AML binding involves at least three hydrogen bonds and contributions from hydrophobic interactions.

MDS also revealed the presence of various intermolecular interactions, including hydrogen bonding, hydrophobic interactions, and water bridges between AML and HSA (Figure 9B). Evidently, hydrogen bonding predominantly contributed to the formation of a stable HSA–AML complex.

In addition, hydrophobic interactions were also crucial for stabilizing HSA–AML complex. The residues Gln390, Arg410, Tyr411, Leu430, Gly434, and Ser489 directly participated in hydrogen-bond formation, whereas Leu453 was involved in a hydrophobic interaction. Notably, the amino acid residues of HSA directly involved in hydrogen bonding and hydrophobic interactions with AML during MDS matched those predicted to be important during Glide XP docking.

## 3. Materials and Methods

### 3.1. Materials

Commercially available HSA (fatty acid-free, ≥96% pure) was purchased from Sigma Aldrich Company (St. Louis, MO, USA). All chemicals used for phosphate-buffer preparation were of biochemical grade.

### 3.2. Sample Preparation

HSA and AML stock solutions were prepared using sodium phosphate buffer (20 mM, pH 7.4). The HSA was diluted in a 20 mM sodium phosphate buffer solution at 4 °C, and its concentration was determined spectroscopically using an extinction coefficient ε_280_ = 36,500 M^−1^cm^−1^ [40]. The concentrations of different solutions were determined on the basis of weight/volume (*w*/*v*) ratio.

### 3.3. Fluorescence-Quenching Measurements

A Jasco spectrofluorometer (FP-8300) equipped with a Peltier-type temperature controller attached to a circulating water bath was used to assess quenching of the fluorescence of HSA at an excitation wavelength of 295 nm, corresponding to Trp-214, in the emission range 300–400 nm at slit widths of 2.5 or 5 nm. Using AML concentrations of 0–25 µM, fluorescence was measured at a HSA concentration of 4 μM. The inner filter effect was corrected in Equation (8), as previously described [11].

(8) $$F_{corr}=F_{obs}\times e^{\left(A_{ex}+A_{em}\right)/2}$$

*F_corr_* and *F_obs_* represent corrected and recorded fluorescence intensities, and *A_ex_* and *A_em_* are the absorbances corresponding to excitation and emission wavelengths.

### 3.4. Synchronous Fluorescence Measurements

Synchronous fluorescence measurements were recorded on a Jasco spectrofluorometer (FP-8300), as previously described [41]. Protein samples were scanned at 260–340 nm and 280–400 nm in the presence and absence of AML, while maintaining differences between excitation and emission wavelengths (∆λ) of 15 and 60 nm for investigating perturbation around Trp and Tyr residues. HSA-synchronous-fluorescence measurements were obtained at AML concentrations of 0 to 25 µM at a HSA concentration of 4 μM.

### 3.5. Three-Dimensional Fluorescence Spectroscopy

The 3D fluorescence spectra of HSA in the absence and presence of AML at 20 μM (1:5 ratio) or 40 μM (1:10 ratio) were acquired by recording emission spectra in the 220–500 nm range using an excitation wavelength range of 230 to 500 nm, and incrementing by 5 nm. For clarity, the entire spectrum is not shown in Figure 6.

### 3.6. UV-Vis Spectra Measurements

UV-Vis spectra of AML at different concentrations were recorded in the 240–340 nm range using a Cary 100 (Varian) double-beam spectrophotometer. UV-Vis spectra of HSA in the presence of various AML concentrations (0–25 µM) were recorded using a cuvette (path length = 1 cm) at a HSA concentration of 10 μM, at a temperature of 298 K and pH of 7.4.

### 3.7. Far-UV Circular Dichroism (far-UV CD) Measurements

A Jasco J-815 spectropolarimeter (JASCO, Tokyo, Japan), a circulating water bath, and a Peltier-type temperature controller were employed to assess the far-UV CD spectra of AML and HSA. The spectropolarimeter was periodically calibrated using D-(10)-camphor sulfonic acid. The far-UV CD spectra of HSA in the presence of AML at molar ratios of 1:5 and 1:10 were obtained using a cuvette of path length 0.1 cm, at a fixed HSA concentration of 4 μM. Measurements were taken in triplicate at a scan speed of 50 nm min^−1^. Raw ellipticity data were later converted into mean residual ellipticity using the following relation:

[*θ*]_λ_ = *θ*_λ_*M*_o_/10*lc* (9)

where *θ*_λ_ represents observed ellipticity (in mdeg) at wavelength; [*θ*] is mean residual ellipticity (in degcm^2^mol^−1^); *M*_o_ is mean residue weight of the protein; *c* is protein concentration (mg/cm^3^); and *l* is path-length (cm). Further, the percentage of α-helix present in HSA under different experimental conditions was calculated using Equation (3)

(10) $$\;\%\alpha -helix=\left(\frac{{-\left[\theta \right]}_{222}-2340}{30300}\right)\times 100$$

where [*θ*]_222_ represents mean residual ellipticity at a wavelength of 222 nm.

### 3.8. Molecular Docking (MD)

A computational check of the possible interaction between AML and HSA was performed using MD, and using “Glide” (Glide, Schrodinger, LLC, New York, NY, USA) as previously described [42]. The crystal structure of HSA (resolution 2.95 Å, PDB Id: 2BXF) was obtained from the PDB database (https://www.rcsb.org/structure/2BXF (accessed on 18 April 2019)) [35] and pre-processed by adding missing hydrogen atoms, converting disulfide bonds to zero bond order, assigning bond orders, and removing heteroatoms that did not interact at least twice with a ligand or protein [43]. After adding missing side chains and loops using “Prime” (Prime, Schrödinger, LLC, NY, USA) [44], the protein was optimized to establish a hydrogen-bond network and then energy was minimized using optimized potentials for liquid simulations 3e (OPLS3e) force field [45] with 0.30 Å RMSD (root-mean-square deviation). MD of the ligand was performed at Sudlow’s sites I and II, located in sub-domains IIA and IIIA, respectively. The dimensions of grid boxes for Sudlow’s site I and II were 24 × 24 × 24 Å located at 4 × −10.5 × 7.3 Å, and 22 × 22 × 22 × Å located at 9.0 × 0.6 × −14.4 Å.

The structure of AML (CID: 16230) was downloaded from the PubChem database (available at NCBI (https://pubchem.ncbi.nlm.nih.gov/compound/16230 (accessed on 18 April 2019))) and prepared using “LigPrep” (LigPrep, Schrödinger, LLC, New York, NY, USA) before conducting MD. The ionization states of AML in the pH range 7.0 ± 2.0 were generated by “Epik” (Epik, Schrödinger, LLC, New York, NY, USA), and, subsequently, the ligand was desalted [46,47]. To ensure a comprehensive exploration of ligand conformational space, a total of 32 stereoisomers were obtained. Energy minimization of the AML conformations was performed using the OPLS3e forcefield to obtain the lowest-energy 3D structure.

### 3.9. Molecular Dynamics Simulation (MDS)

A total of 100 ns MDS using “Desmond” (Desmond, Schrödinger, LLC, New York, NY, USA) with periodic boundary conditions were used to check the stability of the HSA–AML complex [48]. The simulation setup with periodic boundary conditions (PBC) ensured the protein remained at least 10 Å away from the orthorhombic simulation box boundaries. The simulation box was then filled with explicit water using the TIP3P model [49], and, subsequently, counterions were added to neutralize the system. A salt concentration of 0.15 M NaCl was included to maintain an iso-osmotic salt environment. Prior to initiating the simulation, the entire system was energy minimized using the OPLS3e force field and a convergence criterion of 1 kcal/mol/Å over 2000 iterations [45]. Following minimization, the system underwent 100 ns of MDS using an NPT ensemble at 1.013 bars and 300 K. The temperature and pressure were kept constant using Nose–Hoover chains thermostat with a relaxation time of 1 ps [50], and Martyna–Tobias–Klein barostat with isotropic coupling and a relaxation time of 2 ps, respectively [51]. In our simulation study, we used a time step of 2 fs. Constraints were used during this process to keep the positions of the hydrogen atoms stable. These constraints were particularly implemented using the SHAKE algorithm. This method is crucial for avoiding numerical instability, particularly in light of the close proximity of the selected time step to hydrogen vibrational frequencies. We maintain the accuracy and stability of the simulation by adding restrictions through the SHAKE algorithm at intervals of two fs, thereby reducing possible difficulties linked to the forces acting on hydrogen atoms. Non-bonded forces were calculated using a reversible reference system propagator algorithm (r-RESPA) integrator where the short-range forces were updated every step and the long-range forces were updated every three steps. Long-range electrostatic interactions were calculated using the particle mesh Ewald (PME) method [52], and for short-range Coulomb interactions, a cut-off radius of 9.0 Å was used. Energies and structures were recorded every 10 ps and preserved in a trajectory file to enable a simulation interaction diagram to be created. The translational and rotational motions were removed every 2 ps to effectively monitor the system’s internal dynamics and properties.

## 4. Conclusions

In this study, we conducted a comprehensive investigation on the binding of AML to HSA. A variety of techniques were employed, including spectroscopy, thermodynamics, MDS, and MD, to characterize the interaction between the two. This study aimed to identify the binding site on HSA responsible for its interaction with AML and elucidate the mechanism responsible for HSA–AML complex formation. Our findings demonstrate that AML efficiently quenches the fluorescence of HSA by forming a 1:1 stoichiometric complex. The reduction in quenching (*K_SV_*) and binding constant (*K_b_*) at elevated temperatures suggested that the HSA–AML complex exhibits static quenching.

Furthermore, thermodynamic analysis of fluorescence-quenching experiments at varying temperatures showed that HSA–AML complex formation is spontaneous and driven by entropy changes. MD showed AML specifically binds to HSA subdomain IIIA, also known as Sudlow’s site I, through hydrogen-bonding and hydrophobic interactions. FRET analysis provided further evidence of this binding by confirming the proximity of AML and Trp214, which also aligns with the observed quenching effect of HSA. Overall, our findings strongly support efficient binding between AML and HSA and suggest that HSA plays a crucial role in the systemic transport of AML via the plasma. This study provides valuable insights into the binding mechanisms of AML to HSA, enhancing our understanding of the behavior of AML during transport and distribution in vivo and should aid the development of appropriate AML delivery systems.

## Figures and Tables

**Table 1:** Fluorescence-quenching experiments revealed the thermodynamic characteristics of the HSA–AML interaction.

Parameters/Temperature (K)	298	303	310	315
*K_SV_* (M^−1^ )	3.82 × 10^4^	3.25 × 10^4^	2.72 × 10^4^	2.17 × 10^4^
*k_q_* (M^−1^ s^−1^)	6.60 × 10^12^	5.62 × 10^12^	4.70 × 10^12^	3.75 × 10^12^
*K_b_* (M^−1^)	1.89 × 10^4^	1.46 × 10^4^	1.20 × 10^4^	0.99 × 10^4^
*N*	0.93	0.93	0.92	0.93
∆*H*° (kcal mol^−1^)	−6.80			
*T*∆*S*° (kcal mol^−1^)	−0.98	−1.00	−1.02	−1.04
∆*G*° (kcal mol^−1^)	−5.81	−5.80	−5.77	−5.76

**Table 2:** FRET parameters for the interaction between AML and HSA.

	*J* (M^−1^ cm^3^)	*R_o_* (nm)	*r* (nm)	*E*_FRET_
HSA–AML complex	5.99 × 10^−15^	2.26	2.33	0.45

**Table 3:** Three-dimensional fluorescence parameters for the interaction between HSA and AML.

	Peak No.	Peak Position [λex/λem (nm/nm)]	Peak Intensity
HSA only	1	280/335	3736
	2	230/330	1097
HSA + AML (1:5 molar ratio)	1	280/325	3325
	2	230/315	976

## Data Availability

All data is available in the manuscript and in the Supplementary Materials.

## Supplementary Materials

The following supporting information can be downloaded at: https://www.mdpi.com/article/10.3390/molecules28237688/s1.
